# Brown Tumor in a Patient With End-Stage Renal Disease: A Case Report of Secondary Hyperparathyroidism Complications

**DOI:** 10.7759/cureus.103787

**Published:** 2026-02-17

**Authors:** Ahdadi Ahmed, Hamraoui Salima, Assadiki Akram, Zakaria Mimouni, Rachid Marouf

**Affiliations:** 1 Thoracic Surgery, Faculty of Medicine and Pharmacy of Oujda, Mohammed VI University Hospital, Mohammed First University, Oujda, MAR; 2 Thoracic and Cardio-Vascular Surgery, Faculty of Medicine and Pharmacy of Oujda, Mohammed VI University Hospital, Mohammed First University, Oujda, MAR

**Keywords:** brown tumor, chronic kidney disease, hyperparathyroidism, osteodystrophy, parathyroidectomy

## Abstract

The development of a brown tumor is a rare complication of secondary hyperparathyroidism, particularly in patients with chronic kidney disease. Despite advances in the treatment and management of secondary hyperparathyroidism, these tumors can still occur. We report the case of a 29-year-old man with end-stage renal disease treated with hemodialysis, who developed a brown tumor in the ribs. Despite medical treatment including calcium and vitamin D supplementation, and multiple parathyroidectomies, imaging revealed an expansive and sclerotic costal lesion, confirmed as a brown tumor. Diagnosis is based on clinical, biological, and radiological evaluations. Treatments aim to reduce parathyroid hormone (PTH) levels and correct metabolic abnormalities, with parathyroidectomy as an option in cases of medical treatment failure.

## Introduction

Secondary hyperparathyroidism is a frequent and progressive complication in individuals with chronic kidney disease. The decline in renal function disrupts the calcium-phosphate balance, leading to elevated secretion of parathyroid hormone (PTH). Persistently high PTH levels are responsible for disturbances in mineral metabolism and skeletal changes, forming the basis of renal osteodystrophy, as described in major clinical studies on bone involvement in chronic kidney disease [1].

Among the skeletal manifestations of prolonged hyperparathyroidism, brown tumors represent a rare but clinically significant complication. These benign, tumor-like lesions result from exaggerated osteoclastic bone resorption and fibroblastic proliferation driven by excess PTH. Their occurrence and clinical evaluation are closely linked to current definitions and classifications of renal osteodystrophy, which highlight biochemical monitoring as the foundation for diagnosis and management [2]. They may affect various skeletal sites, including the ribs, mandible, pelvis, and long bones. Their diagnosis is often made in patients undergoing regular dialysis and requires careful clinical, radiological, and biochemical assessment. Early recognition is essential to limit disability and improve functional outcomes.

## Case presentation

A 29-year-old man with an eight-year history of hypertension treated with ramipril 5 mg once daily had been followed for nine years for end-stage chronic kidney disease secondary to hypertensive nephropathy, requiring hemodialysis three times per week.

In 2015, the patient was diagnosed with secondary hyperparathyroidism, with a parathyroid hormone (PTH) level of 676 pg/mL, serum calcium of 83 mg/L, phosphate of 72 mg/L, and a vitamin D level of 11.3 ng/mL. Calcium and vitamin D supplementation was initiated.

**Figure 1:**
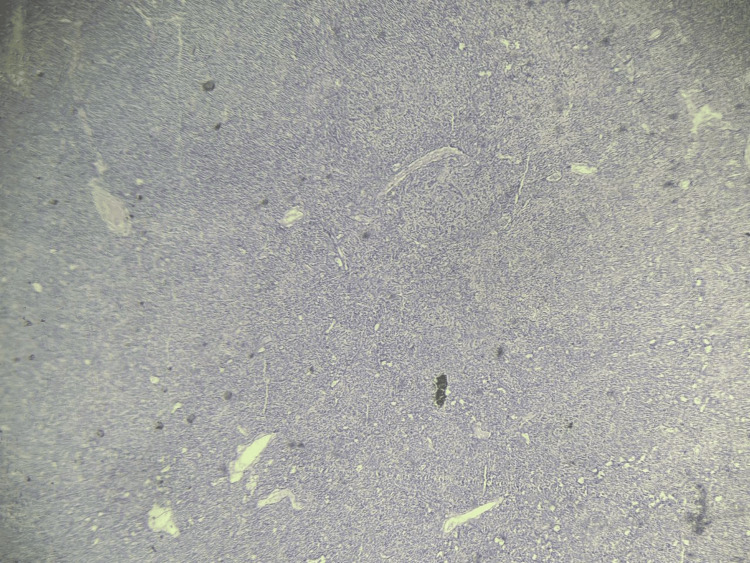
Histological section of the parathyroid gland showing diffuse hypercellularity with reduced intraglandular adipose tissue and focal clusters of water-clear cells with vacuolated cytoplasm (hematoxylin and eosin stain, original magnification ×10).

In 2022, the patient developed severe polyarthralgia associated with left costal pain aggravated by palpation and sciatica. Laboratory investigations showed worsening hyperparathyroidism, with a PTH level of 1,027 pg/mL. Cervical ultrasound revealed bilateral parathyroid nodules. The patient underwent subtotal (three-quarter) parathyroidectomy, and histopathological examination demonstrated parathyroid hyperplasia without evidence of malignancy (Figure 1). The cervical ultrasound was performed at an external facility, and the corresponding images were not accessible for publication at our institution.

**Figure 2:**
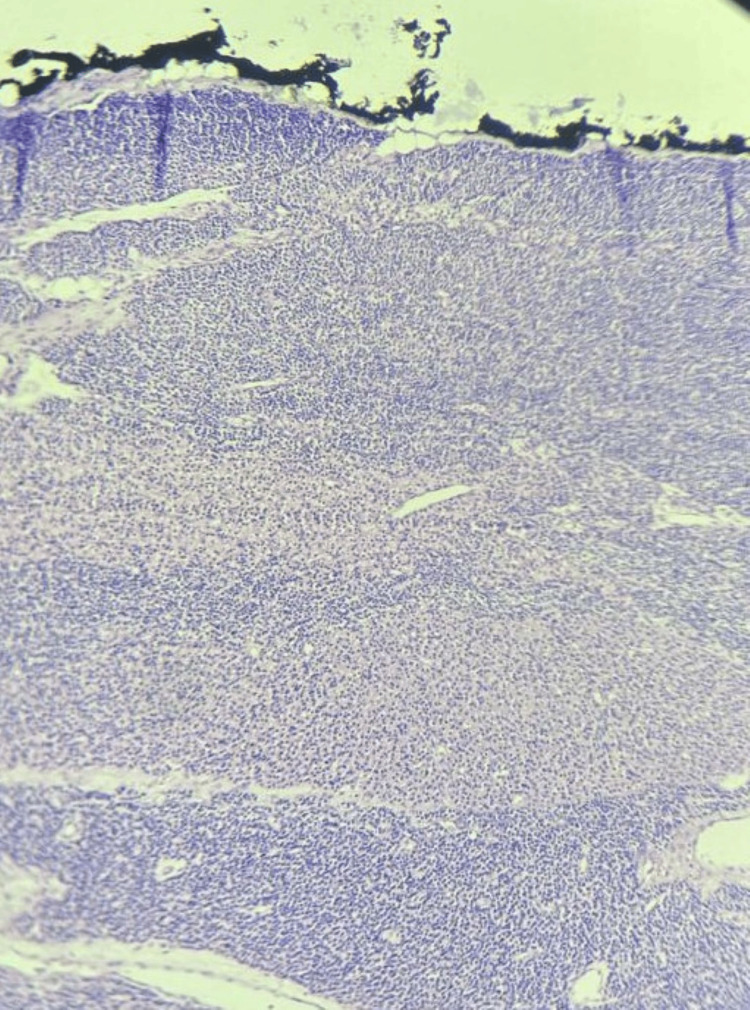
Low-power histological view of the parathyroid gland showing diffuse and nodular hypercellularity with marked reduction of intraglandular adipose tissue (hematoxylin and eosin stain, original magnification ×40).

In 2023, the patient remained symptomatic with persistent hyperparathyroidism (PTH 900 pg/mL). Repeat cervical ultrasound did not demonstrate parathyroid nodules. Parathyroid scintigraphy identified a small oval retrothyroid lesion at the right lower pole, without evidence of ectopic uptake. The patient subsequently underwent right parathyroidectomy, and histological examination again confirmed parathyroid hyperplasia without malignancy (Figure 2). This examination was also performed externally, and the scintigraphic images were not available for retrieval or inclusion in this report.

Postoperatively, the patient was treated with calcium carbonate (Cacit®) 1 g three times daily and alfacalcidol 1 μg/day. At the most recent follow-up in June 2024, laboratory investigations showed normalization of parathyroid function, with a bio-intact PTH level of 13.6 pg/mL, serum calcium of 75 mg/L, phosphate of 9.5 mg/L, and vitamin D level of 32.6 ng/mL.

**Figure 3:**
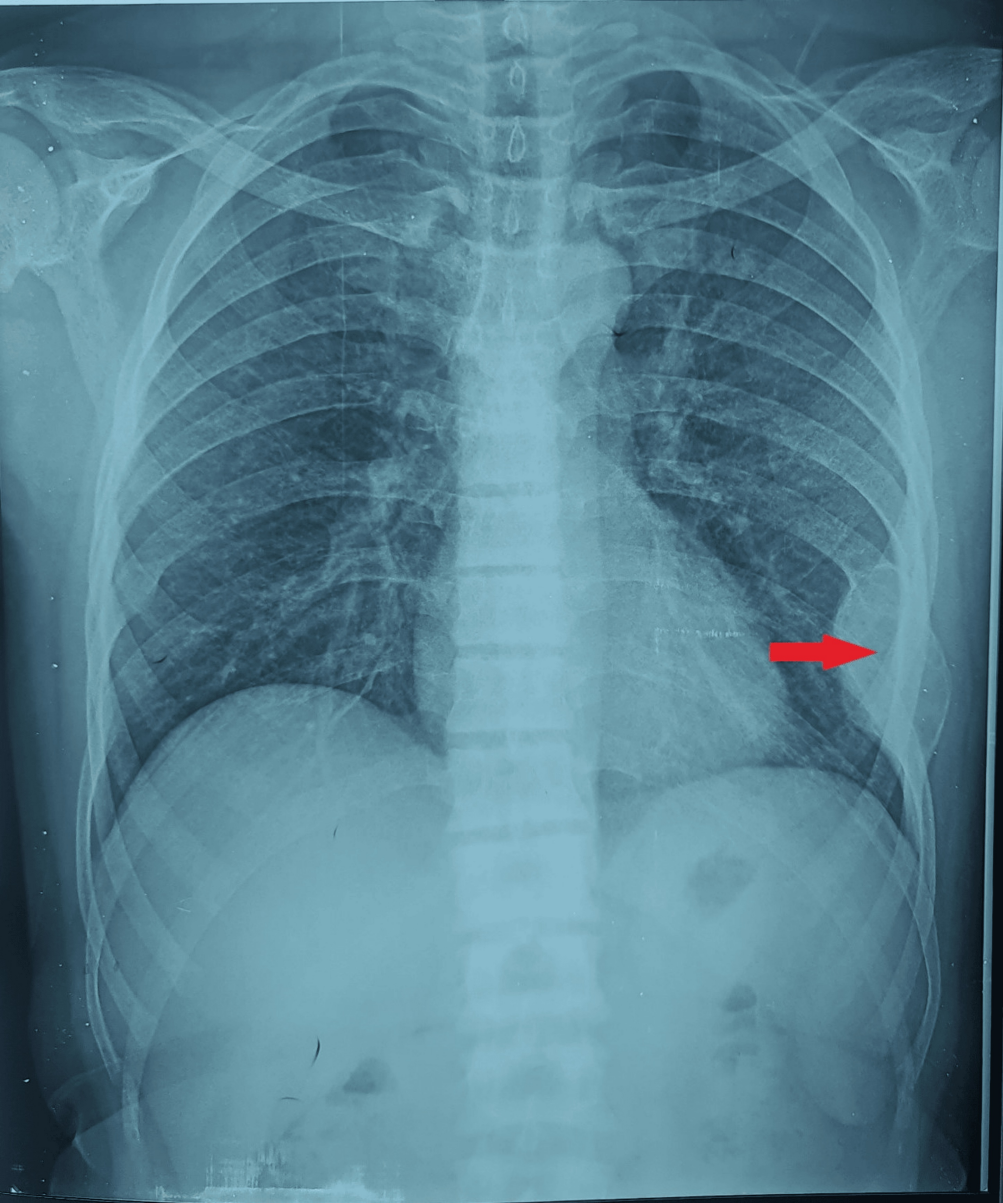
Standard chest X-ray showing a well-demarcated expansile sclerotic lesion of the left eighth rib (red arrow).

**Figure 4:**
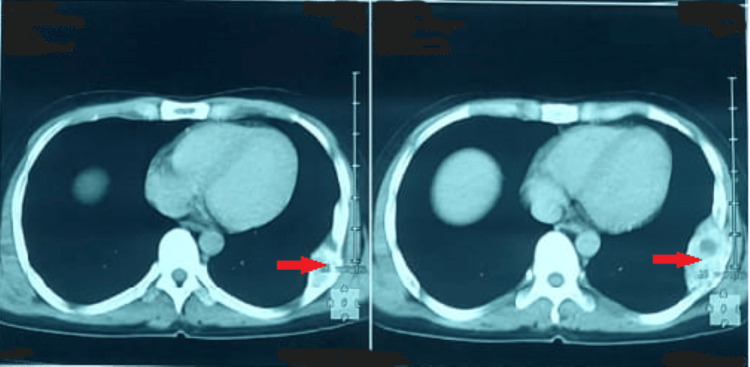
Non-contrast chest CT revealing a 63 × 32 mm sclerotic expansile lesion with preserved cortical margins and no invasive features on the left eighth rib (red arrow).

An osteological assessment was performed. A standard chest radiograph obtained in June 2024 demonstrated a well-defined, expansive, sclerotic lesion involving the middle arch of the left eighth rib (Figure 3). Non-contrast chest computed tomography (CT) confirmed an expansive sclerotic lesion measuring 63 × 32 mm, with well-delineated cortical margins and no suspicious local features (Figure 4).

The combination of secondary hyperparathyroidism with elevated PTH levels and radiologic findings was consistent with a diagnosis of costal brown tumor.

## Discussion

Secondary hyperparathyroidism corresponds to an excessive production of parathyroid hormone (PTH) caused by chronic hypocalcemia and hyperphosphatemia, accompanied by parathyroid gland hyperplasia. It represents a frequent metabolic consequence of chronic kidney disease, as part of renal osteodystrophy [1]. In biological terms, secondary hyperparathyroidism is characterized by markedly elevated PTH levels (>400 pg/mL), associated with hyperphosphatemia and either hypercalcemia, normocalcemia, or hypocalcemia. It is frequently accompanied by a significant increase in total alkaline phosphatase (>240 IU/L) and bone-specific alkaline phosphatase (>25 ng/mL) [2].

Brown tumors, considered a severe form of osteitis fibrosa cystica, are part of this spectrum of renal osteodystrophy [3]. These non-neoplastic lesions result from intense osteoclastic activity, producing focal bone resorption, hemorrhagic areas rich in hemosiderin, and fibroblastic proliferation [4]. They may appear as solitary or multiple lesions and are mostly associated with advanced hyperparathyroidism, occurring in approximately 1.5-1.75% of patients with secondary hyperparathyroidism and 3-4% of those with primary hyperparathyroidism [5]. Although they can develop before or during dialysis, their occurrence after dialysis initiation remains uncommon, with an overall prevalence ranging between 1.5% and 13% in chronic renal failure [6,7]. They can affect any bone, but most commonly involve the pelvis, craniofacial bones, ribs, and mandible [8].

Clinically, brown tumors related to secondary hyperparathyroidism may present with variable manifestations such as bone pain, deformities, pathological fractures, neurological deficits, or symptoms linked to hyperparathyroidism [9,10]. Diagnosis is primarily based on clinical and biochemical findings, with imaging studies supporting the diagnosis. Radiologically, these lesions may appear typical, as expansile, well-circumscribed lytic lesions, or atypical, presenting cortical breaches, irregular destructive defects, and deformities that may complicate diagnosis and require careful evaluation [11]. Histological confirmation is rarely required but may reveal intense osteoclastic resorption, hypervascular fibrous connective tissue containing multinucleated giant cells, hemosiderin deposits, and osteoid matrix [12,13].

Management in patients with chronic kidney disease primarily aims to normalize calcium-phosphate metabolism, lower PTH levels, and prevent extra-skeletal calcifications [14]. A combination of cinacalcet and low-dose vitamin D analogs is frequently recommended [15]. Despite pharmacological progress and improvements in dialysis therapy, many patients ultimately require subtotal or total parathyroidectomy to achieve adequate PTH control [16].

## Conclusions

Secondary hyperparathyroidism is a common metabolic consequence of chronic kidney disease and results in excessive secretion of parathyroid hormone (PTH), ultimately leading to skeletal abnormalities such as brown tumors. These lesions, frequently observed in patients undergoing hemodialysis, warrant careful evaluation because of their potential to cause pain, deformity, and pathological fractures. Diagnosis relies on clinical assessment and biochemical abnormalities, supported by imaging modalities that help characterize both typical and atypical presentations. Management is primarily medical, combining agents such as cinacalcet and vitamin D analogs, while surgical parathyroidectomy remains indicated in cases resistant to medical therapy. Early recognition and optimal control of hyperparathyroidism are essential to limit skeletal complications, improve functional outcomes, and reduce the risk of recurrence.
